# In-plate recapturing of a dual-tagged recombinant *Fasciola* antigen (FhLAP) by a monoclonal antibody (US9) prevents non-specific binding in ELISA

**DOI:** 10.1371/journal.pone.0211035

**Published:** 2019-02-01

**Authors:** Ricardo A. Orbegozo-Medina, Victoria Martínez-Sernández, María J. Perteguer, Ana Hernández-González, Mercedes Mezo, Marta González-Warleta, Fernanda Romarís, Esperanza Paniagua, Teresa Gárate, Florencio M. Ubeira

**Affiliations:** 1 Laboratorio de Parasitología, Facultad de Farmacia, Santiago de Compostela, Spain; 2 Centro Nacional de Microbiología, Instituto de Salud Carlos III, Majadahonda, Madrid, Spain; 3 Laboratorio de Parasitología, Centro de Investigaciones Agrarias de Mabegondo, INGACAL, Abegondo, A Coruña, Spain; Rutgers University, UNITED STATES

## Abstract

Recombinant proteins expressed in *E*. *coli* are frequently purified by immobilized metal affinity chromatography (IMAC). By means of this technique, tagged proteins containing a polyhistidine sequence can be obtained up to 95% pure in a single step, but some host proteins also bind with great affinity to metal ions and contaminate the sample. A way to overcome this problem is to include a second tag that is recognized by a preexistent monoclonal antibody (mAb) in the gene encoding the target protein, allowing further purification. With this strategy, the recombinant protein can be directly used as target in capture ELISA using plates sensitized with the corresponding mAb. As a proof of concept, in this study we engineered a *Trichinella*-derived tag (MTFSVPIS, recognized by mAb US9) into a His-tagged recombinant *Fasciola* antigen (rFhLAP) to make a new chimeric recombinant protein (rUS9-FhLAP), and tested its specificity in capture and indirect ELISAs with sera from sheep and cattle. FhLAP was selected since it was previously reported to be immunogenic in ruminants and is expressed in soluble form in *E*. *coli*, which anticipates a higher contamination by host proteins than proteins expressed in inclusion bodies. Our results showed that a large number of sera from non-infected ruminants (mainly cattle) reacted in indirect ELISA with rUS9-FhLAP after single-step purification by IMAC, but that this reactivity disappeared testing the same antigen in capture ELISA with mAb US9. These results demonstrate that the 6XHis and US9 tags can be combined when double purification of recombinant proteins is required.

## Introduction

The detection of serum antibodies induced by parasitic infections using ELISA methods depends on the availability of enough amounts of specific antigens, either natural or recombinant, to be used in the immunoassays. For this purpose, it would be expected that recombinant antigens were more specific than natural ones, but several studies have shown just the contrary [1–4]. Although this phenomenon has not yet been investigated in detail, some reports pointed out that the presence of contaminants derived from the expression of His-tagged proteins in *E*. *coli* may explain the poor specificity of such antigens [5]. Optimized *E*. *coli* expression [6], acidic washes [7,8], and washing with low amounts of imidazole [9] are methods commonly used to prevent the co-purification of proteins on immobilized metal affinity chromatography (IMAC), thus improving the purity of His-tagged recombinant proteins. Also, disulfide bond formation between the protein of interest and other proteins, as well as nonspecific hydrophobic interactions, can be minimized by inclusion of 2-mercaptoethanol and non-ionic detergents, respectively, in the loading buffer [8]. However, since a relevant fraction of contaminant *E*. *coli* proteins exhibit moderate to strong affinity for metal-chelating resins [9], these methods do not guarantee complete purity of recombinant proteins and may decrease the yield of the purified protein.

Another way to overcome the above problems is grafting a second linear epitope tag recognized by a monoclonal antibody (mAb) into the target sequence of interest, thus allowing re-purification of the protein by affinity chromatography. Currently there are some proprietary tag-mAb pairs that can be used for affinity-purification and for detection of tagged recombinant proteins [10,11]. However, to the best of our knowledge, none of them were used to develop sandwich ELISA methods for serodiagnosis of infectious diseases, a strategy that, if successful, would allow recapturing of the refolded antigen of interest in a single step. To explore this methodology, in the present study we grafted the linear sequence MTFSVPIS, located at the amino terminal region of the gp53 antigen from encapsulated species of *Trichinella* and recognized by the IgG1/κ mAb US9 [12–14], into a *His*-tagged *Fasciola hepatica* recombinant protein (leucine aminopeptidase; FhLAP). FhLAP is a cytoplasmic metalloproteinase isolated from adult flukes [15], which was reported to be able to induce specific antibodies during infection as well as partial protection against reinfection in immunized sheep [16–18]. Although native [19–21] and recombinant [4,22,23] *Fasciola* cathepsins (clades L1, L2 and/or L5) are more adequate as target antigens in ELISA for immunodiagnosis of human and animal infections, for the proof of concept of the present study, we preferred FhLAP since it can be expressed soluble in transformed *E*. *coli* and, consequently, undesirable host proteins are more prone to be present. Moreover, since native FhLAP was previously tested as target antigen for immunodiagnosis of human fascioliasis, this study provides us with the opportunity to evaluate its usefulness to diagnose *F*. *hepatica* infections in domestic ruminants (sheep and cattle).

## Material and methods

### Ethical statement

Blood samples were collected from non-infected and naturally-infected sheep and cattle by veterinarians from the “Centro de Investigaciones Agrarias de Mabegondo” (INGACAL, A Coruña, Spain). The animal experimentation of the present study is part of a research INIA project (RTA2017-00010-C02-01), which was approved by the Ethics Committee of the Consellería do Medio Rural (Xunta de Galicia, Spain). All procedures were carried out in strict accordance with Spanish and EU legislation (Law 32/2007, R.D. 53/2013 and Council Directive 2010/63/EU).

### Collection of biological samples

Sera from non-infected sheep (n = 20) were obtained from animals reared in the *Fasciola*-free environment of the “Centro de Investigaciones Agrarias de Mabegondo” (INGACAL-CIAM), A Coruña (Spain). Sera from *Fasciola*-infected sheep (n = 18) were obtained from the herd of a commercial farm suffering from chronic fascioliasis. All sheep were from an autochthonous Galician breed (‘‘raza gallega”). Confirmation of the presence or absence of infection was done by determination of coproantigens in fecal samples using the MM3-COPRO ELISA [24,25]. In addition, samples of feces from all animals were subjected to sedimentation [26] and flotation [27] procedures to concentrate the helminth eggs, which were then microscopically examined.

Sera from non-infected cattle (n = 49) and naturally *Fasciola*-infected cattle (n = 36) were randomly selected from the collection of sera stored at INGACAL-CIAM. Serum samples from non-infected cattle were collected from animals belonging to Holstein Friesian and “rubia galega” strains reared in the *Fasciola*-free farm from the INGACAL-CIAM. As for non-infected sheep, fecal samples from these animals tested negative in MM3-COPRO ELISA. Serum samples from *Fasciola*-infected cattle were obtained from animals of the strains above, which were sacrificed at local abattoirs and further classified as infected due to the presence of flukes in livers (gold standard). Fecal samples from these animals also tested positive by MM3-COPRO ELISA. As for sheep samples, samples of feces from all animals were examined for the presence of eggs of *F*. *hepatica* and other parasites.

In addition to *F*. *hepatica*, nematodes belonging to the families Trichostrongylidae, Molineidae (*Nematodirus* spp.), Ancylostomatidae, Strongylidae, and Trichuridae (*Trichuris* spp.) were frequently identified in both sheep herds. Regarding cattle samples, individual records at farms revealed that most of them were routinely treated with albendazole during the dry period and vaccinated against infectious bovine rhinotracheitis and bovine respiratory disease (bovine respiratory syncytial virus, parainfluenza virus type 3 and *Mannheimia haemolytica*). Some were also vaccinated at the end of the gestation period against coronavirus, rotavirus and *E*.*coli*, although individual vaccination records were not available. As expected, most of the cows had intestinal nematodes from one or more genera of Trichostrongylidae, Trichuridae (*Trichuris* spp.) and Strongylidae families.

### Bacterial and parasite antigens

The *Fasciola* excretory-secretory antigens (ESAs) used in the MM3-SERO ELISA (see below) were obtained as previously reported [28,29]. Briefly, live adult flukes were collected from the bile ducts of naturally infected cows and washed, first in sterile saline solution containing antibiotics (penicillin/streptomycin) and glucose (2 g/L), at 38°C, and then in RPMI 1640 cell culture medium supplemented with 20 mM HEPES, 0.3 g/L L-glutamine, 2 g/L sodium bicarbonate and antibiotics, at 38°C under 5% CO_2_ in air. The flukes were then transferred to 75-cm^2^ tissue culture flasks and maintained in culture medium (3 mL/fluke) at 38°C under 5% CO_2_ in air. After 24 h incubation, the medium containing the secreted antigens was removed and centrifuged at 10,000 g for 20 min at 4°C in the presence of protease inhibitors (SigmaFast Protease Inhibitor Tablets, Sigma-Aldrich, Madrid, Spain). The supernatant was then passed through a 0.45-μm pore filter disk, concentrated in an Amicon 8050 ultrafiltration cell (Amicon, Inc., Beverly, MA) equipped with a YM10 membrane (10 kDa molecular weight cut-off), dialyzed against PBS, sterilized by filtration and, finally, stored at -80°C until required. The protein concentration was measured using the Micro BCA Protein Assay Kit (Pierce; Thermo Fisher Scientific, Barcelona, Spain).

### Production and purification of mAbs US9 and MM3

Hybridoma cells secreting mAbs US9 and MM3 were obtained as previously described [13,24]. The secreting hybridoma cells were grown intraperitoneally in pristan-primed BALB/c mice, and the anti–*Trichinella* and *F*. *hepatica* IgG1/κ antibodies were purified from the ascitic fluid by caprylic acid/ammonium sulfate precipitation [30].

### Cloning of the FhLAP gene

Complementary DNA (cDNA) synthesis was carried out with the Marathon cDNA amplification kit (Clontech, Palo Alto, CA) employing 1 μg of adult *F*. *hepatica* mRNA extracted by Fast Track mRNA Isolation Kit (Invitrogen, San Diego, CA) and following the protocols described in Muiño et al. [31]. The FhLAP gene was amplified by Touchdown-PCR using a set of primers based on the sequence gb|AY644459: FhLAP-Fc: 5'-ATGGCGGCGTTGGCTGTGGGCGTGTCT-3' FhLAP-Rc: 5'-CTATTTGAATCCCAGTCGTGGTAGTACGTAACGGCTGGCGAAC-3'. A combination of a DNA polymerase and a hot start enzyme Taq DNA Polymerase (Advantage mix polymerase kit, Takara Bio USA Inc., Mountain View, CA) was used following the provider’s instructions. The *F*. *hepatica* cDNA was used as template at a 1/250 dilution. Then the amplified FhLAP gene (1562 bp) was cloned into the vector pGEM-T vector (Promega Biotech Ibérica SL, Madrid, Spain) following standard protocols [32]. Positive recombinant plasmids were selected by standard PCR with the universal primers D and SP6. Further, the plasmid FhLAP DNA extracted from the gel (QIAquick Gel Extraction, Qiagen; Qiagen Iberia SL, Madrid, Spain) was automatically sequenced, using fluorescence-base labeling with ABI PRISM system (Perkin Elmer, Langen, Germany), to rule out any sequences errors.

### Production and subcloning of the US9-FhLAP chimeric gen

A chimera (US9-FhLAP) containing the complete FhLAP gene and the amino acids (MTFSVPIS) corresponding to the epitope of the mAb US9 [14] was constructed. The chimera assembly was performed by standard PCR, using standard polymerase (Biotools, Madrid, Spain) and the primers US9-FhLAP-F: 5'-GGATCC-ATGACATTTTCAGTTCCTATTTCCATGGCGGCGTTGG-3'. US9-FhLAP-R: 5'-AAGCTT-CTATTTGAATCCCAGTCGTGG-3'. Plasmid pQE30-US9-FhLAP DNA (10 LAP1) was used as template at a 1/1,000 dilution to amplify the chimera. Directional cloning in the pQE expression vector (Qiagen) was performed using the BamH I and Hind III restriction endonucleases (Roche Molecular Systems, Inc.) and following standard protocols [32]. Positive recombinant plasmids were selected by standard PCR with the universal primers pQE-forward and pQE-reverse. Further, the plasmid US9-FhLAP DNA extracted from the gel (QIAquick Gel Extraction, Qiagen) was automatically sequenced using fluorescence-based labeling with ABI PRISM system (Perkin Elmer, Langen, Germany) to rule out any sequences errors. Besides the universal pQE forward and reverse primers, the following four new primers (two designed in the amino and carboxy terminal regions and two in middle of the LAP sequence) were used in the sequencing procedure: FhLAP AMINO FWD: 5'-ATGGCGGCGTTGGCTGTG-3', FhLAP CENTRAL REV: 5'-CATAAGTGATACCTTTTCCAATCA-3', FhLAP CENTRAL FWD: 5'-GAGCCGCCCAATCCAACCGAGG-3', FhLAP CARBOXY REV: 5'-CTATTTGAATCCCAGTCGTGG-3'. Recombinant pQE30-US9-FhLAP was transformed into XL1 Blue cells (Agilent, Santa Clara, CA), which were mixed with 20% of sterile glycerol and stored at -80°C.

### Obtaining and purification of rUS9-FhLAP

The M15 [pREP4] *E*. *coli* strain (Qiagen) was transformed with 10 ng of recombinant pQE30-US9-FhLAP DNA. A colony of transformed cells was grown in 1 L of LB medium at 37°C with shaking (200 rpm/min) until reaching an OD_600_ of 0.5 and the rUS9-FhLAP protein expression induced by the addition of 0.5 mM IPTG to the cell culture and further incubation for 6 h at 22°C with shaking (120 rpm/min). Then, the cells were harvested by 20 min centrifugation at 5,000 *g* and stored frozen during 24 h at -30°C. Afterwards, the pellet was resuspended in 50 mL of B-PER Bacterial Protein Extraction Reagent (Thermo Fisher Scientific) supplemented with lysozyme and DNase I, as indicated by the supplier, pipetted up and down until the suspension was homogeneous and incubated with shaking (150 rpm/min) for an additional 30 min at RT. To separate soluble proteins from the insoluble ones the cell lysate was centrifuged at 15,000 *g* for 15 min and the supernatant containing the soluble rUS9-FhLAP was purified by IMAC chromatography with the HIS-Select Nickel Affinity Gel (Sigma-Aldrich) under non-denaturing conditions.

To carry out the IMAC procedure, the column containing the HIS-Select Nickel Affinity Gel was pre-equilibrated with PBS and the solution containing soluble rUS9-FhLAP (pre-diluted 1/4 with PBS containing 10 mM imidazole) was added with the aid of a peristaltic pump (5 mL/min). Then, the column was washed with 5 volumes of PBS containing 10 mM imidazole and the protein eluted with PBS containing 250 mM imidazole under UV monitoring. Finally, the protein was concentrated by ultrafiltration in an Amicon Stirred Ultrafiltration Cell equipped with a Filtron Omega Series membrane (10 kDa nominal molecular weight limit; Pall Corporation, Port Washington, NY), filtered by 0.22 μm, supplemented with 5% glycerol and stored at -30°C until later use in ELISA. Prior to addition of glycerol, the protein concentration was measured by Micro BCA Protein Assay Kit (Thermo Fisher Scientific). Additionally, non-transformed M15 cells were grown at 37°C for 8h without addition of IPTG, harvested by centrifugation and lysed with B-PER reagent as indicated as above. Then, the B-PER solution containing the soluble proteins was diluted 4-fold with PBS containing 5 mM imidazole and subjected to IMAC chromatography under the same conditions as above, except that PBS containing 5 mM imidazole was used for column washes.

### SDS-PAGE and Western-blotting (WB) analysis

The soluble protein fraction extracted after induction with IPTG of transformed, and non-transformed, M15 cells (see above), and the protein fractions purified by IMAC were separated in 10–20% linear gradient polyacrylamide gels, as previously described [33]. The separated proteins were stained with BlueSafe (Nzytech, Lisboa, Portugal) or transferred to PVDF membranes (Immobilon-P, Millipore Ibérica SA, Madrid, Spain) in a Trans-Blot SD transfer cell (Bio-Rad Laboratories, Richmond, CA, USA) and blocked with PBS with 0.05% Tween 20 and 1% dry skimmed milk (PBS-T-SM) for 2 h at RT on an orbital shaker. Membranes were then incubated with mAb US9 (1/2,000) followed by HRP-conjugated goat anti-mouse IgG antibody (Bio-Rad, 1/3,000) after a washing step (three times with PBS-T for 5 min each), or with HRP-conjugated mouse anti-polyhistidine (Sigma-Aldrich, 1/100,000). Each antibody was incubated for 1 h at RT on an orbital shaker. Finally, the membranes were washed again, and the bands were developed using 3,3-diaminobenzidine tetrahydrochloride tablets (Sigma-Aldrich), following the supplier’s instructions.

### ELISA procedures

#### Capture ELISA with mAb MM3 (MM3-ELISA)

The MM3-ELISA was used as reference method to compare the sensitivity and specificity of this test with the results obtained using rUS9-FhLAP as target antigen in indirect and capture ELISAs (see below). This method, which uses the mAb MM3 to capture *Fasciola* L-cathepsins secreted by adult flukes (L1, L2 and L5 [4,31]), is very similar to the previously described MM3-SERO ELISA [34] with some modifications. Briefly, polystyrene microtiter plates (Greiner BioOne 96 well 8 X F12 strips, high binding plates) were coated with purified mAb MM3 (100 μL/well at 5 μg/mL in PBS), incubated ON at 4°C, washed three times with PBS and blocked with 200 μL/well of 1.5% sodium caseinate in PBS for 1 h at RT. Aliquots of 100 μL of *F*. *hepatica* ESAs at 1 μg/mL in PBS or PBS only were added to each well in odd (Ag+) and even (Ag-) plate rows, respectively. The plates were incubated for 2 h at RT and then washed three times with PBS, before 100 μL of each serum sample (from sheep or cattle) diluted 1/100 in PBS-T-SM was added to each Ag+ and Ag- well in duplicate. The plates were then incubated for 30 min at RT with shaking at 750 rpm, washed five times with PBS-T, and bound IgG antibodies were detected with either HRP-conjugated mouse anti-sheep/goat IgG monoclonal antibodies (Sigma–Aldrich, 1/30,000 in PBS-T-SM), or HRP-conjugated sheep anti-bovine IgG1 polyclonal antibodies (Bio-Rad, 1/6,000 in PBS-T-SM). The plates were then washed, as above, and incubated for 20 min at RT with 100 μL/well of the enzyme substrate OPD (SigmaFast OPD, Sigma-Aldrich). Finally, the optical density (OD) was measured at 492 nm. The OD values for each sample were calculated as OD1-OD2, where OD1 is the value for the Ag+ well, and OD2 is the value for the Ag- well. Two cut-off values for sheep and cattle were considered to maximize specificity. Cut-off 1 was calculated as the sum of the maximal OD value obtained for negative sera from each species plus one SD [4] and cut-off 2 was determined by means of Receiver Operating Characteristic (ROC) analysis for 100% specificity.

#### Capture ELISA with mAb US9

Polystyrene microtiter plates (Greiner BioOne 96 well 8 X F12 strips, high binding plates) were coated with purified mAb US9 (100 μL/well at 5 μg/mL in PBS), incubated ON at 4°C, washed three times with PBS and blocked with 200 μL/well of 1.5% sodium caseinate in PBS for 1 h at RT. Aliquots of 100 μL of a previously calculated optimal concentration of IMAC-purified rUS9-FhLAP (15 μg/mL) in PBS-T-SM, or PBS-T-SM only, were added to each well in odd (Ag+) and even (Ag-) plate rows, respectively. The plates were incubated for 30 min at RT with shaking at 750 rpm and then washed five times with PBS-T, before 100 μL of each serum sample (from sheep or cattle) diluted 1/100 in PBS-T-SM was added to each Ag+ and Ag- well in duplicate. The plates were then incubated for 30 min at RT with shaking at 750 rpm, washed five times with PBS-T and specific sheep or bovine IgG was detected as described above. The OD values for each sample and the cut-off values were calculated as for the MM3-ELISA.

#### Indirect ELISA with rUS9-FhLAP

The wells of ELISA plates were coated with 100 μL of a previously calculated optimal concentration of IMAC-purified rUS9-FhLAP protein (3 μg/mL) in PBS, incubated for 2 h at 37°C, washed three times with PBS and blocked with 200 μL/well of 1.5% sodium caseinate in PBS for 1 h at RT. Aliquots of 100 μL of each serum sample (from sheep or cattle) diluted 1/100 in PBS-T-SM were added to each well of the plates in duplicate and incubated for 30 min at RT with shaking at 750 rpm. The plates were then washed five times with PBS-T, and bound IgG antibodies were detected as described above. The OD values for each sample and the cut-off values were calculated as for the MM3-ELISA.

#### Indirect competitive ELISA with rUS9-FhLAP

In order to determine the possible reactivity of sera with the US9 epitope included in the rUS9-LAP chimeric sequence, an indirect competitive ELISA was developed with the cattle serum samples in parallel with the above indirect rUS9-FhLAP ELISA. As inhibitor, the synthetic peptide MTFSVPIS (US9-recognizing epitope) was used. The mAb US9, diluted 1/1,000, was used as a control to calculate the optimal inhibitory concentration of the US9 synthetic peptide, which was tested in the range 0.5 μM to 1 mM. Complete inhibition of US9 binding was achieved at a peptide concentration of 0.0625 mM; consequently, the previous peptide dilution (0.125 mM) was selected to be used for inhibition of sera in indirect competitive ELISA. This assay was performed as described above for indirect rUS9-FhLAP ELISA but using cattle sera prediluted 1/100 in PBS-T-SM containing 0.125 mM of the US9 peptide and incubated for 30 min at RT before addition to the ELISA wells.

#### Indirect ELISA with IMAC-retained M15 proteins

The wells of ELISA plates were coated with 100 μL of IMAC-retained M15 soluble proteins (4 μg/mL) in PBS, incubated ON at 4°C, washed three times with PBS, and blocked with 200 μL/well of 1.5% sodium caseinate in PBS for 1 h at RT. Aliquots of 100 μL of sera from non-infected cattle diluted 1/100 in PBS-T-SM were added to the wells of the plates in duplicate and incubated for 30 min at RT with shaking at 750 rpm. The plates were then washed five times with PBS-T, and bound IgG antibodies were detected with HRP-conjugated sheep anti-bovine IgG1 polyclonal antibodies as described above. The OD values for each sample were calculated by subtracting the non-specific binding obtained in the absence of cattle serum (OD = 0.042).

#### LPS immunoassays

The wells of ELISA plates were coated with 100 μL of a PBS solution containing LPS from *E*. *coli* serotype O55:B5 (Sigma-Aldrich) at 10 μg/mL, incubated for 2 h at 37°C, washed three times with PBS, and blocked with 200 μL/well of 1.5% sodium caseinate in PBS for 1 h at RT. Aliquots of 100 μL of each serum sample from non-infected cattle, diluted 1/100 in PBS-T-SM, were added to each well of the plates in duplicate, and incubated for 30 min at RT with shaking at 750 rpm. The plates were then washed five times with PBS-T, and bound IgG antibodies were detected as described above for MM3-ELISA. Finally, the OD was measured at 492 nm.

To detect the presence of LPS from M15 cells in the wells of ELISA plates coated with rUS9-FhLAP, or mAb US9 plus rUS9-FhLAP, the plates were incubated with 100 μL/well of a goat anti-lipid A LPS polyclonal antibody (ThermoFisher Scientific, Rockford, USA) diluted 1/100 in PBS-T-SM and incubated for 30 min at RT with shaking at 750 rpm. The plates were then washed five times with PBS-T, and bound IgG antibodies were detected with HRP-conjugated mouse anti-sheep/goat IgG monoclonal antibodies (Sigma–Aldrich, 1/30,000 in PBS-T-SM). Finally, the OD was measured at 492 nm. Wells of an ELISA plate coated with *E*. *coli* O55:B5 LPS (see above) were used to adjust the adequate dilution of anti-lipid A LPS polyclonal antibody, which gave an mean OD value of 0.52 at a 1/100 dilution under these conditions.

### Bioinformatics tools

Theoretical isoelectric points, instability indices, aliphatic indices and grand average of hydropathicity (GRAVY) values were calculated using the ProtParam bioinformatics tool from Expasy (https://www.expasy.org/resources).

### Statistical analysis

The Spearman correlation coefficients (*r*) were calculated using GraphPad InStat 3.05 (GraphPad Software, Inc., La Jolla, CA) and OriginPro v 7.5 (OriginLab Corporation, Northamptom, CA) software packages. Differences were considered significant at p <0.05. The ROC curves were calculated using the MedCalc v. 17.7.2 software (MedCalc Software, Ostend Belgium).

## Results

### Cloning and expression of the rUS9-FhLAP chimeric protein

**Fig 1:**
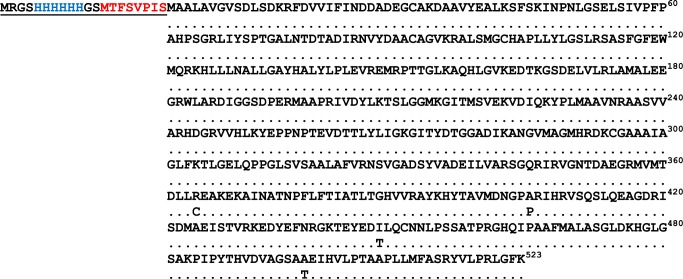
Alignment of gb| AAV59016 (parental) and gb| MF945962 (this study) recombinant FhLAPs from *F*. *hepatica*. Showed are the differences in the amino acid sequences between both proteins as well as the amino terminal region containing the two inserted tags (6XHis, blue) and US9 (MTFSVPIS, red), and the extra residues (including two GS dipeptides) introduced during the cloning procedure.

The alignment of the amino acid sequence corresponding to the ORF of the FhLAP protein cloned in this study (gb| MF945962) and the sequence reported by Acosta et al. (unpublished results; gb| AAV59016.1) is shown in Fig 1. The extra amino terminal region (underlined), which includes the corresponding 6XHis tag (blue) and the US9 epitope (red), is also displayed. Considering the US9 epitope sequence alone, the ProtParam bioinformatics tool indicated that such sequence is hydrophobic in nature, with an aliphatic index of 85 and a GRAVY value of 1.188 (highly hydrophobic). However, considering the complete amino acid sequence inserted in the amino terminal region of rUS9-FhLAP, i.e., the first 20 residues in Fig 1 (underlined), the aliphatic index decreased to 34.0 and the GRAVY value was -0.735 (moderately hydrophilic). In addition to the initial residue M, one R and two dipeptides (GS) flanking the 6XHis and the US9 tags were also introduced in the recombinant protein because of the cloning procedure in the pQE30 vector excised with BamH I and Hind III restriction enzymes.

Comparing the cDNA sequences gb| MF945962 and gb| AAV59016, we observed that both have the same extension (1569 bp) a very high identity (99.99%) with only 8 nucleotide changes (C^31^T, C^78^A, G^93^A, T^1090^C, C^1210^G, C^1251^T, C^1337^T, A^1489^G). Moreover, the translation of the nucleotide sequence gb| MF945962 revealed the existence of only 4 amino acid changes located in the carboxy terminal region (C^364^R; P^404^A; T^446^I and T^497^A) with respect to the parental sequence gb| AAV59016 (see Fig 1).

**Fig 2:**
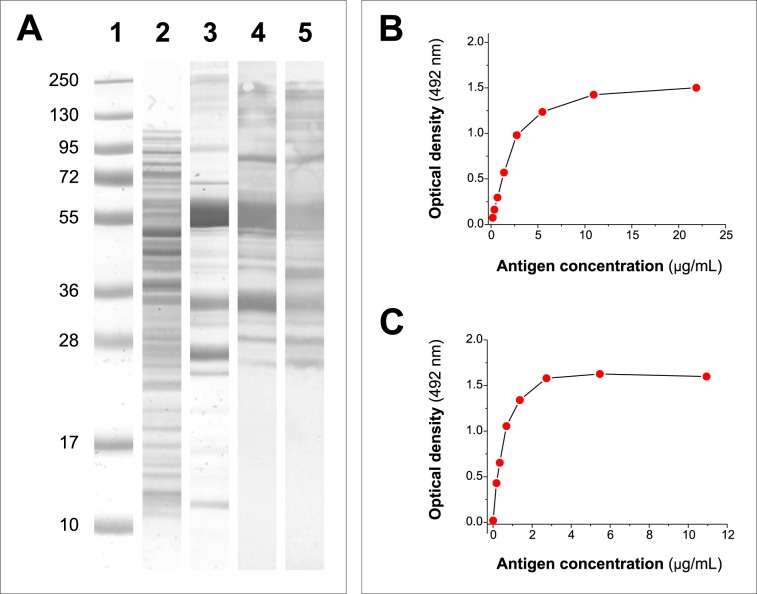
Analysis of the expression of the chimeric recombinant protein rUS9-FhLAP in *E*. *coli* and optimization of its use in ELISA. (A) SDS-PAGE and WB analysis of rUS9-FhLAP expression. Lane 1: MW markers. Lane 2: Coomasie blue staining of the soluble protein fraction obtained after induction with 0.5 mM IPTG. Lane 3: Coomasie blue staining of rUS9-FhLAP after purification by IMAC. Lane 4: WB analysis of rUS9-FhLAP revealed with mAb US9 as primary antibody. Lane 5: WB analysis of rUS9-FhLAP revealed with HRP-conjugated anti-polyhistidine. (B) ELISA OD values obtained testing different concentrations of rUS9-FhLAP (range: 0.7–22 μg/mL) as target antigen in capture ELISA with mAb US9. (C) ELISA OD values obtained testing different concentrations of rUS9-FhLAP (range: 0.3–11 μg/mL) as target antigen in indirect ELISA. The analysis was carried out with a serum from a *Fasciola*-infected sheep as primary antibody.

As deals with the expression of rUS9-FhLAP, the WB analysis shown in Fig 2A revealed that the protein is correctly expressed in *E*. *coli*, appearing as a major band with an approximate Mr of 60 kDa (theorethical MW = 58,665.25 Da). Also, several minor bands of different MW (lane 3) can be observed, which probably correspond to truncated proteins and/or *E*. *coli* contaminants copurified during the IMAC procedure. The presence of rUS9-FhLAP truncates could be confirmed in WB revealing the proteins with mAb US9 (lane 4) and a mAb recognizing the poly-His tag (Fig 2A, lane 5). Moreover, the excellent recognition of both the whole protein and rUS9-FhLAP truncates by mAb US9 indicated that the positioning of the US9 epitope between the poly-His tag and the FhLAP sequence is adequate for the objective of the study. In addition, we investigated if the rUS9-FhLAP is also correctly recognized in ELISA by a serum from a *Fasciola*-infected sheep. The data in Fig 2B and 2C showed that the sheep serum reacted with the recombinant antigen in a dose-dependent manner and that the ELISA signal reached a plateau at antigen concentrations over 10 μg/mL for US9-ELISA and over 3 μg/mL for indirect ELISA.

### Recognition of rUS9-FhLAP by sera from infected and non-infected ruminants

As indicated above, a single-step affinity-purification of soluble recombinant antigens produced in *E*. *coli* by IMAC may involve the presence of variable amounts of contaminant proteins from the host which may have negative consequences on the specificity of indirect ELISAs. To investigate whether this inconvenient can be avoided by in-plate recapturing of the rUS9-FhLAP antigen by mAb US9, we compared the recognition of such antigen in indirect and capture US9-ELISA by sera from non-infected or naturally *F*. *hepatica*-infected sheep and cattle. Each serum was tested simultaneously in indirect ELISA using rUS9-FhLAP coupled to the wells of ELISA plates, and in two capture ELISAs, US9-ELISA and MM3-ELISA (reference method). Also, since the study was designed to investigate the effect of contaminating proteins present in antigen preparations affecting the specificity of the assays, two cut-off values (cut-off 1 and cut-off 2; see Material and Methods section) that guarantee maximum specificity were used.

**Fig 3:**
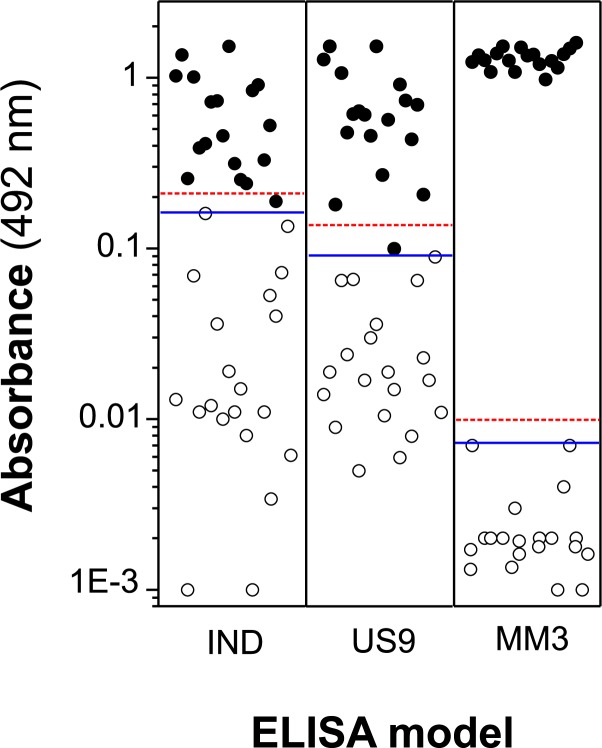
ELISA OD values obtained testing sera from *Fasciola*–free (open circles) and *Fasciola*–infected (closed circles) sheep. Each point in the figure represents the mean OD value (two replicates) obtained for one individual serum sample. IND = sera tested in indirect ELISA with rUS9-FhLAP antigen as target. US9 = sera tested in capture ELISA with mAb US9 coupled to ELISA plates and rUS9-FhLAP as target antigen. MM3 = sera tested in capture ELISA with mAb MM3 (MM3-ELISA; reference test). Horizontal lines represent the cut-off values (red dashed line: cut-off 1; blue line: cut-off 2). Cut-off 1 and cut-off 2 values for each ELISA were as follows: OD = 0.205 and 0.16 (IND); OD = 0.114 and 0.089 (US9); OD = 0.009 and 0.007 (MM3).

Considering sera from sheep (Fig 3) and cut-off 1, 17/18 (94.4%) true positive samples were obtained by indirect ELISA, while 100% of samples from infected sheep tested positive using the less-restrictive cut-off 2 value, i.e., calculated by ROC analysis. The same percentages were also obtained analyzing the sera by US9-ELISA, but the serum testing negative using the respective cut-off 1 in indirect ELISA and in US9-ELISA was not the same.

Although most sheep sera were correctly classified using either indirect ELISA or capture US9-ELISA, if we consider the differences obtained between the OD values for each positive sera and the corresponding cut-off values, the best results were obtained, as expected, with MM3-ELISA, followed by US9-ELISA. In this sense, it should be noted that, for both cut-offs, these differences were greater than 0.9 for our reference method (MM3-ELISA), while only 4/18 (22.2%) of sera reached these values with the other two ELISA methods. These data strongly suggest that FhLAP is recognized during infection of sheep by *F*. *hepatica* but that this protease provokes a lesser antigenic stimulus than L-cathepsins, probably due to two facts: i) unlike FhLAP (cytoplasmatic), *Fasciola* L-cathepsins are the most abundant secreted proteins, thus being permanently in contact with host immune cells, and ii) during its biological cycle, *Fasciola* produces different L-cathepsins [35] that have high sequence identity and thus may reciprocally boost the production of antibodies.

**Fig 4:**
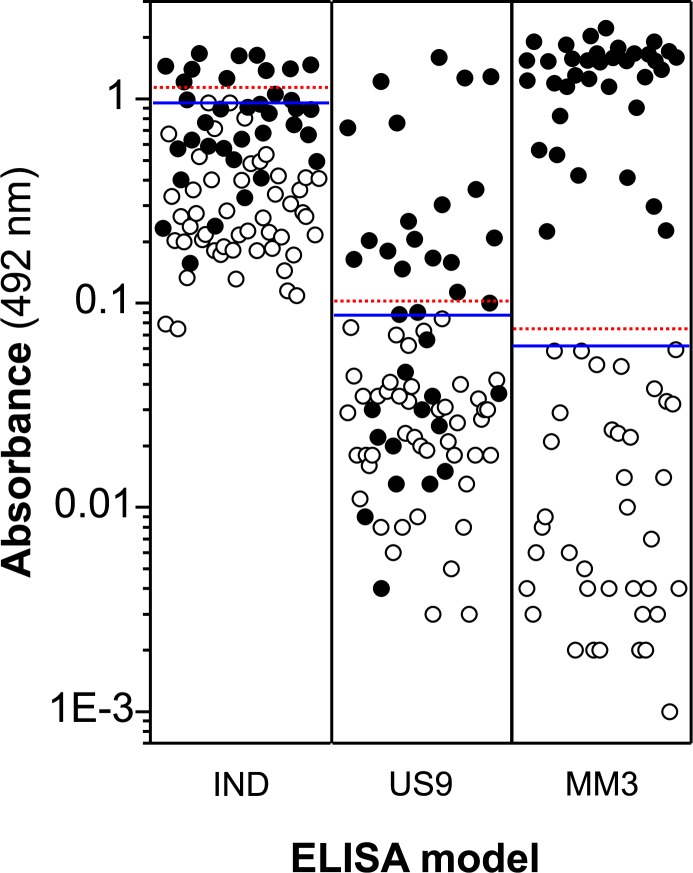
ELISA OD values obtained testing sera from *Fasciola*–free (open circles) and *Fasciola*–infected (closed circles) cattle. Each point in the figure represents the mean OD value (two replicates) obtained for one individual serum sample. IND = sera tested in indirect ELISA with rUS9-FhLAP antigen as target. US9 = sera tested in capture ELISA with mAb US9 coupled to ELISA plates and rUS9-FhLAP as target antigen. MM3 = sera tested in capture ELISA with mAb MM3 (MM3-ELISA; reference test) and *Fasciola* ESAs as target antigen. Horizontal lines represent the cut-off values (red dashed line: cut-off 1; blue line: cut-off 2). Cut-off 1 and cut-off 2 values for each ELISA were as follows: OD = 1.174 and 0.963 (IND); OD = 0.111 and 0.084 (US9); OD = 0.075 and 0.059 (MM3).

As for sheep, all sera from infected and non-infected cattle were correctly classified using the MM3-ELISA regardless of the two cut-off values investigated (Fig 4). However, the data in Fig 4 also revealed that the antigens present in the rUS9-FhLAP preparation are strongly recognized by a large number of non-infected cattle sera in indirect ELISA. In this sense, we observed that 33/49 (67.3%) of sera from non-infected cattle produced OD values ≥0.2 (range OD = 0.069–0.963) after subtracting the corresponding non-specific binding value of the plate (OD = 0.06; without serum). As expected, the high reactivity of these sera limited the number of infected cattle sera testing positive in indirect ELISA, as only 10/36 (27.8%) and 13/36 (36.1%) samples were classified as positive using cut-off 1 and cut-off 2, respectively.

Unlike in the indirect ELISA, capture of the rUS9-FhLAP antigen by mAb US9 drastically decreased the OD values obtained with non-infected cattle sera. As can be observed in Fig 4, as for the MM3-ELISA, the US9-ELISA provided OD values below 0.1 (range 0.0–0.084). However, the results in Fig 4 also demonstrated that, contrary to L-cathepsins, only part of the infected cattle produced specific antibodies to FhLAP. Specifically, 18/36 (50%) and 20/36 (55.5%) of infected cattle sera tested positive by US9-FhLAP ELISA using cut-off 1 and cut-off 2, respectively. Nevertheless, these results were better than those obtained by indirect ELISA (see above).

**Fig 5:**
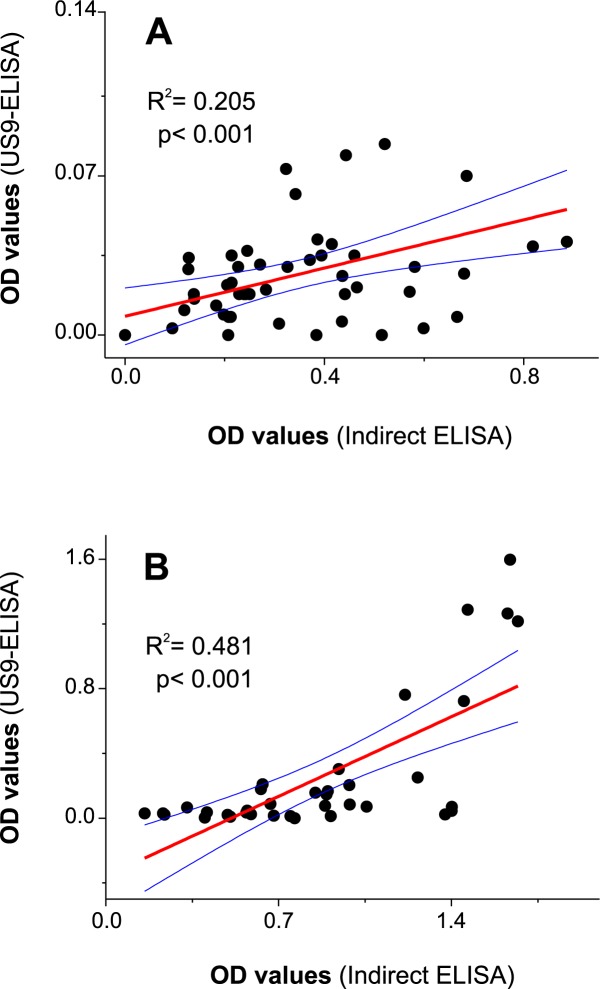
**Least squares linear regression analysis obtained comparing non-infected (A) and *Fasciola*-infected (B) cattle sera OD values obtained in indirect ELISA and US9-ELISA.** Each point represents the paired OD values obtained for each serum by both methods. Blue lines represent the 95% upper and lower confidence levels.

Although indirect ELISA and capture US9-ELISA immunoassays use the same rFhLAP antigen, given that they are different in design, a doubt arises concerning the concordance between classification of positive and negative sera by both methods. To clarify this aspect, we performed a linear regression analysis between the OD values obtained for non-infected or *Fasciola*-infected cattle by both methods. The results in Fig 5 showed that OD values obtained for non-infected (Fig 5A) and infected (Fig 5B) cattle sera by both methods have very significant correlation (P<0.001). However, as indicated by the relatively low R^2^ values (0.20 and 0.48 for non-infected and infected cattle sera, respectively), particularly for non-infected cattle sera, a considerable amount of sera produced discordant OD values comparing both ELISA methods.

**Fig 6:**
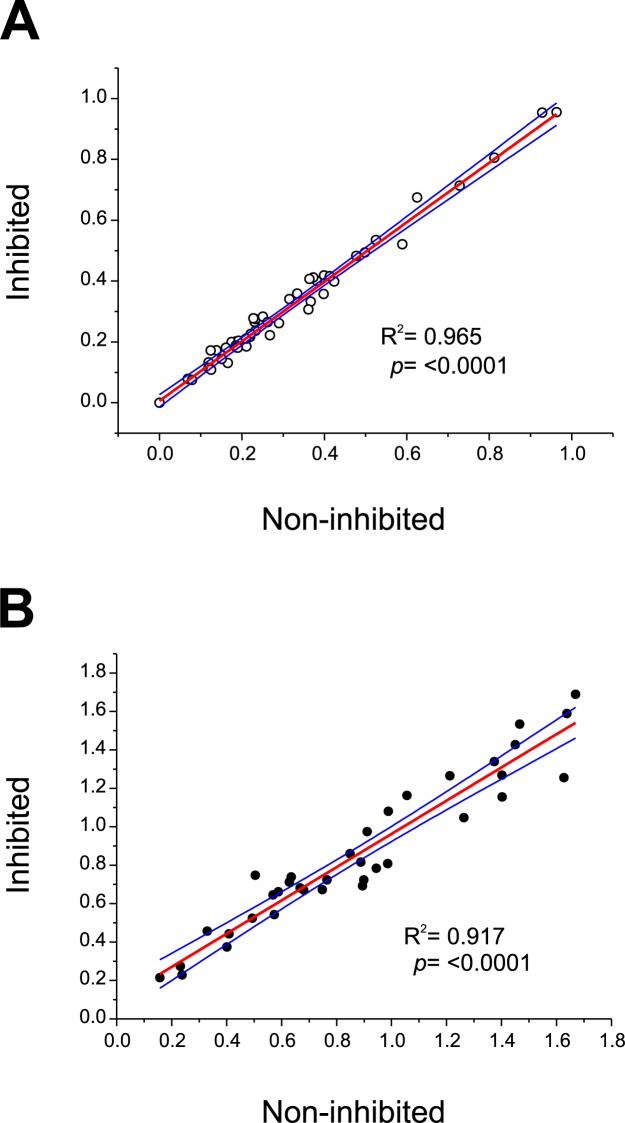
**Least squares linear regression analysis comparing non-inhibited and inhibited (MTFSVPIS peptide) sera from non-infected cattle (A) or sera from *F*. *hepatica* naturally-infected cattle (B).** Each point represents the paired OD values obtained for each serum in indirect ELISA using rUS9-FhLAP as target antigen. Blue lines represent the 95% upper and lower confidence levels.

In our US9-ELISA, the MTFSVPIS epitope is blocked by the paratope of mAb US9, thus avoiding its recognition by sera from infected or non-infected animals. However, since this epitope is freely available when the rUS9-FhLAP antigen is used in indirect ELISA, it could hypothetically be recognized by cross-reactive antibodies present in sera from infected or non-infected animals. To discard this possibility, in parallel with the indirect ELISA performed with the rUS9-FhLAP antigen (see above), we carried out an inhibition ELISA using a synthetic peptide derived from the gp53 *Trichinella* protein containing the MTFSVPIS sequence recognized by mAb US9. As expected, the incubation of cattle sera with the peptide did not change the reactivity of non-infected (Fig 6A) and infected cattle sera (Fig 6B), as was deduced from the excellent correlation coefficients between OD values of non-inhibited and inhibited sera (R^2^ = 0.965 and R^2^ = 0.917 for non-infected and infected cattle sera, respectively).

**Fig 7:**
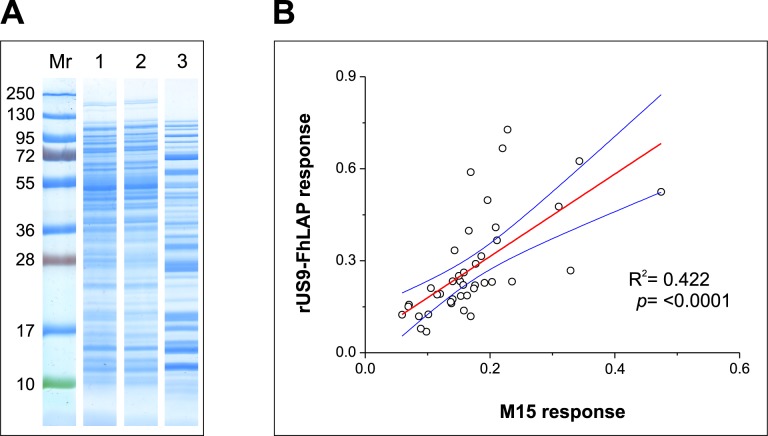
Analysis of soluble *E*. *coli* proteins binding to Nickel Affinity Gel columns. (A) SDS-PAGE analysis of M15 *E*. *coli* soluble proteins present in the starting sample (lane 1), in the non-retained fraction (lane 2), and in the eluted fraction (lane 3, concentrated). The relative mobility of MW standards is shown on the left. (B) Least squares linear regression analysis comparing OD values obtained in indirect ELISA for sera from non-infected cattle (n = 35) using as target IMAC-purified proteins from non-transformed (M15 response; X-axis) or transformed (rUS9-FhLAP response; Y-axis) M15 *E*. *coli* cells. Each point represents the paired OD values obtained for each serum in the indirect ELISAs. Blue lines represent the 95% upper and lower confidence levels.

In an attempt to identify probable target(s) of cross-reactivity showed by cattle sera, we first investigated whether this could be due to soluble M15 *E*. *coli* proteins that could bind to the chromatographic column and co-elute during IMAC purification of rUS9-FhLAP. To this end, the protein profiles of soluble M15 proteins, before and after IMAC-purification, were firstly revealed by SDS-PAGE and protein staining with BlueSafe (Fig 7A). Then, the eluted protein fraction was used as target antigen in indirect ELISA to investigate whether it is recognized by sera from non-infected cattle (Fig 7B). The results in Fig 7 clearly showed that: i) a broad MW range (10–200 kDa) of soluble proteins from non-transformed M15 *E*. *coli* bond to the IMAC column (Fig 7A), ii) these proteins are recognized in indirect ELISA by sera from non-infected cattle (mean OD = 0.17±0.08SD; OD range: 0.07–0.47), and iii) the reactivity observed in indirect ELISA testing sera from non-infected cattle (n = 35) significantly correlated (R^2^ = 0.42; p<0.0001) with that obtained using IMAC-purified rUS9-FhLAP (Fig 7B). These data, together with the fact that the IMAC-purified M15 protein fraction was able to inhibit 50–70% of the reactivity of selected sera from non-infected cattle in an indirect ELISA with rUS9-FhLAP (not showed), clearly indicates that native *E*. *coli* proteins bind and co-elute with recombinant proteins during IMAC purification, and thus constitute a relevant source of non-specific binding by natural antibodies present in sera from cattle. Moreover, since such reactivity was not observed when testing the same sera in WB (not showed), it seems that the target of cattle antibodies are mainly conformational epitopes.

**Fig 8:**
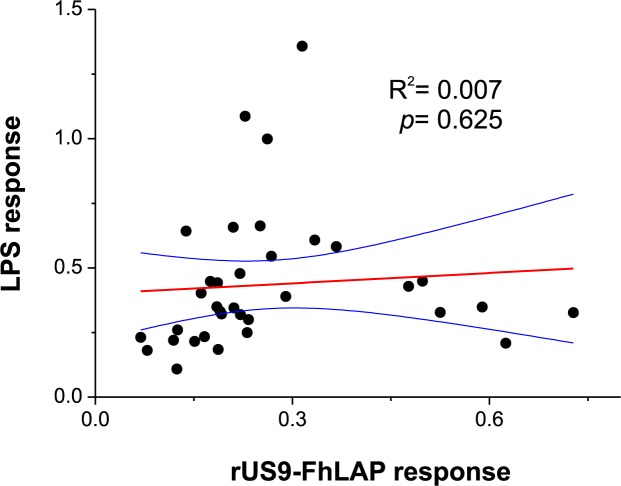
Least squares linear regression analysis between OD values obtained in indirect ELISA testing sera from non-infected cattle in response to rUS9-FhLAP (rUS9-FhLAP response; X-axis) and LPS from *E*. *coli* serotype O55:B5 (LPS response; Y-axis). Each point represents the paired OD values obtained for each serum. Blue lines represent the 95% upper and lower confidence levels.

Another possible target for cross-reactive natural antibodies in cattle is LPS [36]. To investigate whether a putative contamination by M15 LPS of rUS9-FhLAP coupled to ELISA plates might be targeted by serum antibodies from non-infected cattle, we checked for the presence of lipid A in the wells of ELISA plates using polyclonal anti-lipid A antibodies. In addition, we investigated a possible correlation between OD values obtained with sera from non-infected cattle against rUS9-FhLAP and against *E*. *coli* O55:B5 LPS, both in indirect ELISA. As deals with the presence of lipid A, the anti-lipid A polyclonal antibodies produced low OD values in wells of ELISA plates coated with rUS9-FhLAP (OD = 0.12) and no signal in the wells coated with mAb US9 plus rUS9-FhLAP. Moreover, the results in Fig 8 showed no correlation (p = 0.62) between OD values obtained for non-infected cattle sera tested against rUS9-FhLAP and LPS, both in indirect ELISA. These data suggests that, in our experimental conditions, contamination by LPS is not a relevant target for the cross-reactivity observed testing sera from cattle.

## Discussion

Epitope tagging is a genetic engineering technique in which a protein encoded by a cloned gene is fused to an epitope recognized by a known antibody [37,38]. This strategy allows rapid affinity-purification of recombinant proteins, but it can also be used for protein detection in WB, flow cytometry and ELISA [39]. Typically, epitope tags (commonly, 5–16 residues in length) are selected among linear sequences [10,40] and have a negative GRAVY index (i.e., hydrophilic) assuming that hydrophilic tags are exposed at the surface and thus are more accessible to antibodies. Also, in most studies tags have been placed at the amino or carboxy terminal regions in the belief that the loss of function will be minimal, although this is not a general rule applicable to any protein (reviewed in [37]).

In our study, we engineered a *F*. *hepatica* recombinant FhLAP by incorporating two affinity tags in tandem (6XHis and MTFSVPIS) at its amino terminal region aimed to improve its purification free of contaminants by mAb US9 after a first purification by IMAC. While the classical 6XHis is the most commonly used tag for single-step purification of recombinant proteins, allowing purities of up to 95% with 90% recovery of the tagged protein [8], the remaining contaminants may be of great importance when these antigens are used as target in immunodiagnosis or in any other situation where a homogeneous product is required. The advantage of using a dual tag could be evidenced in our study, in which the rUS9-FhLAP purified in a single step by IMAC showed strong reactivity in indirect ELISA with many sera from non-infected ruminants (mainly from cattle), but disappeared when these antigens were re-purified by mAb US9 in capture ELISA. Typical metal-binding contaminants during IMAC purifications include non-protein components (e.g., phospholipids, nucleic acids and LPS), but also host cell proteins like outer membrane proteins, stress-responsive proteins (folding machinery) and ribosomal subunit proteins [41], some of which require more than 80 mM imidazole for elution [9].

Although the molecular identification of *E*. *coli* proteins recognized by sera from sheep and cattle goes beyond the objectives of the present study, the results of the SDS-PAGE showing the presence of bands that were not recognized by US9 and anti-His antibodies in WB (Fig 2A), revealed the presence of contaminant *E*. *coli* proteins in the IMAC-purified rUS9-FhLAP. Likewise, a great diversity of *E*. *coli* proteins with affinity for Ni(II) ions was also observed among soluble proteins obtained from non-transformed M15 cells (Fig 7A) using PBS plus 5mM imidazole as loading and washing buffer. It is also noticeable that false positive results using IMAC-purified recombinant proteins is not limited to the rUS9-FhLAP antigen of present study, as similar observations were also reported using the more antigenic *Fasciola* recombinant cathepsins L as target in ELISA after expression in *Pichia pastoris* and further purification by IMAC [2]. Moreover, recent studies in our laboratory showed that the number of false positives was less relevant when the antigens (procathepsins L) were expressed in *E*. *coli* as inclusion bodies, as these aggregates are first washed by centrifugation and most of contaminant host soluble proteins are eliminated prior to denaturation and IMAC affinity-purification [4].

In addition to proteins, LPS was also recognized as a frequent contaminant during IMAC purification of recombinant protein expressed in *E*. *coli* [42]. Although the *E*. *coli* M15 cells used in this study derive from a K12 *E*. *coli* strain (https://www.qiagen.com/fr/resources/resourcedetail?id=abc8b958-d415-4b91-88a1-e5f90d0a1884&lang=en), which lacks the highly antigenic O antigen part of LPS [43], it still retains LPS components, including the lipid A [44], which is potentially antigenic. As in previous studies [36], our data in Fig 8 showed that sera from cattle free of *F*. *hepatica* present relevant levels of anti-LPS (*E*. *coli* serotype O55:B5) antibodies. However, the absence of correlation between such antibody response and that obtained against rUS9-FhLAP excludes *E*. *coli* lipid A as a source of cross-reactivity in our study.

Regarding the physicochemical characteristics of the MTFSVPIS sequence recognized by mAb US9, it is important to note that, unlike most of previously reported epitope tags [10], it has intrinsic positive GRAVY (1.188), which denotes its hydrophobic nature and thus would advise against its use to tag some recombinant proteins. Nevertheless, it should be underlined that its insertion into the rFhLAP sequence was performed in tandem after the 6XHis tag and that 5 additional residues were also incorporated into the amino terminal region due to the cloning procedure. Consequently, twenty extra residues were incorporated into the amino region of the rFhLAP sequence (^1^MRGSHHHHHHGSMTFSVPIS^20^) with a GRAVY = -0.735, which is in the range of many other epitope tags [10]. It is also noticeable that the US9 epitope in the chimeric rUS9-FhLAP has a similar position as in the parental mature sequence of gp53 from *T*. *spiralis* (^1^STDNENVAMKEMTFSVPIS^19^, GRAVY = -0.289; gb|CAD86781.1) and *T*. *britovi* (^1^STDNENAAMKEMTFSVPIS^19^, GRAVY = -0.416; gb|CAD86782.1) [13], which anticipated the good recognition of rUS9-FhLAP by mAb US9. Moreover, the presence of GS dipeptides before the 6XHis and the US9 epitope probably also facilitates the recognition by mAb US9, as “GS” linkers are commonly used as flexible linkers to engineer recombinant fusion proteins [45,46] and to link epitopes within chimeric multiepitope recombinant antigens [47,48]. Finally, it should also be considered that, although the MTFSVPIS sequence belongs to a nematode protein, its recognition by sera from infected animals in capture ELISA is not possible since such epitope is not available after binding to the capture antibody (US9).

By testing sera from humans infected with *F*. *hepatica*, Marcilla et al. [17] reported that a *F*. *hepatica* native FhLAP may have potential interest in serodiagnosis of human fasciolosis. However, compared with cathepsins L (MM3-ELISA) our results clearly demonstrated that, at least in ruminants, the responses to this antigen are discrete and, consequently, cannot be recommended for developing immunodiagnostic tests.

In summary, and as a proof of concept, in this study we demonstrated for the first time that the introduction of a hydrophobic epitope tag recognized by a mAb into a recombinant antigen from *Fasciola* can be used for in-plate recapturing of the antigen allowing the design of new and specific capture ELISAs. This strategy may be advantageous over indirect ELISAs by at least two reasons: i) unlike in indirect ELISA, all antigen molecules remain oriented in the space by action of the capture mAb, and ii) an individual control, i.e., a well containing mAb molecules and the blocking protein, can be used for each serum, thus improving detection of individuals having antibodies reacting with either the capture mAb or the proteins used to block the ELISA plates [34]. Further studies are being carried out in our laboratory to investigate if the same strategy can be performed with other *Fasciola* antigens, such as whole or selected fragments of cathepsins L, to develop robust immunodiagnostic tests without the necessity of obtaining live parasites in abattoirs.
